# Activation of Ftz-F1-Responsive Genes through Ftz/Ftz-F1 Dependent Enhancers

**DOI:** 10.1371/journal.pone.0163128

**Published:** 2016-10-10

**Authors:** Amanda Field, Jie Xiang, W. Ray Anderson, Patricia Graham, Leslie Pick

**Affiliations:** Department of Entomology and Program in Molecular & Cell Biology, University of Maryland, College Park, Maryland, 20742, United States of America; Instituto Gulbenkian de Ciencia, PORTUGAL

## Abstract

The orphan nuclear receptor Ftz-F1 is expressed in all somatic nuclei in *Drosophila* embryos, but mutations result in a pair-rule phenotype. This was explained by the interaction of Ftz-F1 with the homeodomain protein Ftz that is expressed in stripes in the primordia of segments missing in either *ftz-f1* or *ftz* mutants. Ftz-F1 and Ftz were shown to physically interact and coordinately activate the expression of *ftz* itself and *engrailed* by synergistic binding to composite Ftz-F1/Ftz binding sites. However, attempts to identify additional target genes on the basis of Ftz-F1/ Ftz binding alone has met with only limited success. To discern rules for Ftz-F1 target site selection in vivo and to identify additional target genes, a microarray analysis was performed comparing wildtype and *ftz-f1* mutant embryos. Ftz-F1-responsive genes most highly regulated included *engrailed* and nine additional genes expressed in patterns dependent on both *ftz* and *ftz-f1*. Candidate enhancers for these genes were identified by combining BDTNP Ftz ChIP-chip data with a computational search for Ftz-F1 binding sites. Of eight enhancer reporter genes tested in transgenic embryos, six generated expression patterns similar to the corresponding endogenous gene and expression was lost in *ftz* mutants. These studies identified a new set of Ftz-F1 targets, all of which are co-regulated by Ftz. Comparative analysis of enhancers containing Ftz/Ftz-F1 binding sites that were or were not bona fide targets in vivo suggested that GAF negatively regulates enhancers that contain Ftz/Ftz-F1 binding sites but are not actually utilized. These targets include other regulatory factors as well as genes involved directly in morphogenesis, providing insight into how pair-rule genes establish the body pattern.

## Introduction

Highly conserved cascades of regulatory genes control embryonic development of diverse animal species. These regulatory genes are often members of large families, encoding DNA binding transcription factors (TFs) that activate or repress expression of larger sets of downstream or target genes that are directly involved in cell growth and differentiation. Understanding mechanisms used by embryonic TFs to select genomic binding sites is key to understanding their function. Protein-protein interactions play a major role in target site selection, particularly for transcription factors with weak monomeric DNA binding specificity. Additionally, the activity of ubiquitously expressed TFs can be limited by restricted expression of a key partner protein, thus relieving constraints on specific expression domains for the remaining members of a regulatory complex.

The Hox family of TFs regulates segmental identity and controls diverse processes in all metazoans. Hox proteins share a highly conserved sequence-specific DNA binding domain, the homeodomain [1]. Despite the unique biological activities seen for individual Hox proteins in vivo, their homeodomains all bind very similar DNA sequences [2, 3] (reviewed in [4]. This ‘Hox paradox’ was thought to have been resolved by the identification of binding partners for Hox proteins, including the divergent homeodomain-containing protein, Extradenticle (Exd), which provides increased specificity to Hox DNA binding [5–9]. However, it is surprising that so many Hox proteins can heterodimerize with the same partner and maintain diverse regulatory specificity. Recent reports suggest that Hox-Exd specificity is achieved through the different protein complexes’ distinguishing between different low affinity binding sites [10]. In contrast, the Hox protein Fushi Tarazu (Ftz), which functions as a pair-rule segmentation gene in *Drosophila*, has acquired a unique partner, the orphan nuclear receptor Ftz-F1.

*ftz* arose as a homeotic gene as a result of an ancient duplication of a *ftz/Antennapedia* ancestral gene but has taken on exclusive pair-rule function in *Drosophila*. Ftz lost the ability to functionally interact with Exd in *Drosophila* and gained the ability to specifically interact with Ftz-F1 [11–18] (reviewed in [19, 20]. Ftz and Ftz-F1 form a stable complex in vitro and in vivo and bind synergistically to DNA sequences that contain binding sites for both Ftz and Ftz-F1 (composite sites) to coordinately activate transcription. Ftz-F1 has a longer and more specific DNA binding site than do Hox proteins, increasing the specificity of binding for the Ftz/Ftz-F1 heterodimer in vivo. Thus, the Hox protein Ftz evolved a method for achieving specificity different from other Exd-dependent Hox proteins, possibly obviating the requirement for Ftz and its partner to distinguish between high and low affinity sites, as do Hox-Exd complexes. At the same time, despite the fact that *ftz* and *ftz-f1* mutants display indistinguishable pair-rule mutant phenotypes, Ftz-F1 is expressed in all somatic nuclei at the blastoderm stage and is a strong transcriptional activator in vitro [11, 17, 21]. This suggested that its activity is limited to cells which co-express Ftz (Ftz+ cells). In *Tribolium castaneum*, Ftz-F1 is expressed in stripes and has pair-rule function that appears to be independent of Ftz, suggesting that constraints on Ftz-F1 expression in *Drosophila* were relaxed as a result of its obligate interaction with Ftz, expressed in stripes [22]. However, the possibility that Ftz-F1 regulates a subset of genes in *Drosophila* in cells lacking Ftz (Ftz^-^ cells) has not been ruled out.

Previous attempts to characterize how Ftz and/or Ftz-F1 regulate their targets have met with some success in identifying new target genes and associated Ftz/Ftz-F1-responsive cis-regulatory elements (CREs). One of the best characterized targets of Ftz and Ftz-F1 is *ftz* itself, which is autoregulated through independent enhancers in the upstream element [23, 24]. Similarly, Ftz and Ftz-F1 coordinately regulate the expression of *engrailed* (*en*) in seven stripes through binding to composite sites in an intronic CRE [12]. A computational screen based on predicted Ftz/Ftz-F1 binding sites in the genome identified *apt* and *Sulf1* as Ftz/Ftz-F1-responsive genes, while a screen based on expression patterns of candidate targets identified *drm*, *noc*, and *5-HT2A* [25, 26]. Of the seven targets characterized in these studies, including *ftz* itself and *en*, Ftz/Ftz-F1-responsive CREs were identified for only three of these–*ftz*, *en*, and *drm*. Each contains consensus Ftz and Ftz-F1 binding sites but no other sequence features distinguished these enhancers.

Here we identified Ftz-F1-responsive genes by microarray analysis. The top candidate genes were tested as possible Ftz/Ftz-F1 targets by examining their expression patterns in relation to *ftz* expression and expression in *ftz* and *ftz-f1* mutants. Interestingly, all candidates that were dependent on Ftz-F1 also required Ftz, supporting the model that Ftz and Ftz-F1 are obligate partners in gene regulation in the early embryo. To determine whether these are direct Ftz/Ftz-F1 targets, the DNA surrounding these targets was examined for Ftz binding and candidate Ftz-F1 binding sites. Six of the eight candidate enhancers tested directed reporter gene expression in patterns resembling the endogenous gene, and expression was dependent upon Ftz. Analysis of motifs suggested that Deaf-1 and Zeste may function as co-activators of Ftz/Ftz-F1 targets while DNA-binding proteins GAGA factor (GAF) and Dichaete may inhibit Ftz/Ftz-F1 genomic binding.

## Materials and Methods

### Fly stocks and molecular genetics

Flies were maintained at 25°C on a standard diet. The *ftz* mutant was *ftz*^*9H34*^/*TM3Ser*, *hb-lacZ*, with expression of β-galactosidase used to identify mutant embryos. Enhancer-reporter constructs were constructed by PCR isolation of ~ 1kb regions of genomic DNA inserted into the following sites of attB*lacZ*, upstream of a basal promoter and *lacZ*: *ken*—EcoRI/ XbaI, *aay*—EcoRI/XbaI, *mid*–HindIII/XbaI, *tal*—HindIII/XbaI, *5-HT2A* –EcoRI/XbaI, *trn*–HindIII/XbaI, *hh*–XbaI/HindIII, *Antp*–HindIII, XbaI, and *blot*–XbaI/NotI. The PhiC31 integration system was used to insert transgenes into the genomic attP site VK00022 in chromosome II. Transgenic fly lines were generated by Rainbow Transgenic Flies, CA and BestGene, CA and were maintained as homozygotes. One transgenic line, containing the *blot* enhancer construct, was homozygous lethal but, when crossed into a *ftz* mutant background, was homozygous viable. In this *ftz* background, a rare phenotype was observed in which part of the right dorsal thorax was missing and only the left wing was present. To examine transgene expression in *ftz* embryos, virgin females *w; ftz*^*9H34*^/*Tm3Sb* were crossed with *w; P[enhancer-lacZ]*/*P[enhancer-lacZ]*; *Dr*/*Tm3Sb* males. From this cross, *w; P[enhancer-lacZ]*/*+; ftz*^*9H34*^*/Tm3Sb* males and females were crossed to generate *w*; *P[enhancer-lacZ]*/*P[enhancer-lacZ]*; *ftz*^*9H34*^*/Tm3Sb*. Offspring were self-crossed to analyze expression in a *ftz* background. Embryos derived from *ftz-f1* germline clones (referred to as *ftz-f1*mutants) were generated with the autosomal FLP-DFS technique [27–29] using *ftz-f1*^*19*^ [30, 31]. Briefly, *yw hsFLP;FRT*^*2A*^*ftz-f1*^*19*^*/TM3Sb* virgin females were crossed with *w; FRT*^*2A*^ *ovo*^*D*^*/TM3Sb* males. Females were allowed to lay eggs for 1 day in vials and their progeny were heat-shocked for 2 hours at 37°C in a circulating water bath on the third and fourth days after egg laying. Subsequently, embryos derived from the females of genotype *yw hsFLP;FRT*^*2A*^*ftz-f1*^*19*^/ *FRT*^*2A*^ *ovo*^*D*^ (identified as non-*Sb* females) were analyzed. All of the *FRT*^*2A*^ *ovo*^*D*^ recombinant chromosomes were associated with a fully penetrant DFS phenotype such that all eggs laid by these females are derived from germline recombination events. For the control for the microarray experiment, *yw hsFLP;FRT*^*2A*^*/ FRT*^*2A*^ virgins were crossed to *w; FRT*^*2A*^ *ovo*^*D*^*/TM3Sb* males and subjected to the same heat shock and selection protocol in parallel.

For in situ hybridization, standard protocols were followed [32, 33] with one modification—in place of Proteinase K treatment, embryos were heated at 95°C for 5 minutes. Primer sequences for probes available upon request. Standard protocols were followed for antibody staining [34]. For reporter constructs, anti-β-galactosidase antibody (Cappel, 1:2000) was used. Stained embryos were visualized using DIC on a Leica DMRB microscope, a Zeiss Discovery V12, or a Leica SP5X Confocal microscope.

### Microarray

*ftz-f1* or control females (see above) were mated to *w*^*1118*^ males in collection cages at 25°C and allowed to lay eggs for up to 2 hours. Eggs were dechorionated in 3% sodium hypochlorite for 3 minutes and then covered with halocarbon oil and aged. Embryos were visualized under phase contrast optics at 100-200x magnification. Individual embryos were pooled into groups of roughly equivalent developmental stages and kept out of the light path of the microscope as much as possible. To generate pools of embryos at specific stages, each pool was monitored closely and selected by visual inspection at late cellularization, stage 5, at the onset of gastrulation, stage 6, or at mid germband extension, stage 8. Embryos were manipulated using a small needle. As each embryo reached the desired stage of development, it was transferred to 100 μl of TRIZOL on ice. Approximately 100 embryos were collected per time point. Individual collections were stored at -80°C. Each experiment was done in triplicate. Total RNA was extracted using the Qiagen RNEasy kit according to standard protocols. Samples were processed using the Affymetrix one-cycle cDNA synthesis protocol prior to hybridization to Affymetrix Drosophila 2.0 expression arrays.

Affymetrix drosophila2 genechip CEL files were imported into BioConductor/R [35] using the ReadAffy function of the affy package [36] and assigned to developmental stage (5,6,8), wildtype or *ftz-f1* mutant condition (0,1) and batch number (processing and hybridization batch). Using 3 replicates of 3 stages and 2 states of *ftz-f1* gave a total of 18 genechip arrays. All arrays were normalized by the expresso function using quantiles normalization, only perfect match, and median polish summary method. This generated the normalized expression set used for all further data analysis. The normalization procedure produced log_2_ expression results, and the fold change between the average of any two data sets s1 and s2 was calculated as $\frac{s2-s1}{\left|s2-s1\right|}2^{\left|\text{s}2-\text{s}1\right|}$. To identify differentially expressed genes showing a response to the presence or absence of functional Ftz-F1, the microarray analysis of variance package, maanova [37], was run in oneColor mode fitting a mixed effect ANOVA model using the formula ŷ = Stage+Ftz.F1+Batch. Batch represents the groups of RNA that were processed and hybridized on the same day and was treated as a random or non-repeatable term in the model. The function matest was run with a permutation count of 100 to compute the p-value for the Ftz.F1 model term. p-values were further controlled for the N discovery rate using the Q-value method of the adjPval function [38]. Probesets having an FDR adjusted p-value < 0.05 were considered potential target genes.

### Motif Analysis

401bp windows surrounding the peak Ftz binding position for all intervals [39] were merged and the underlying genomic sequences were extracted from a repeat masked copy of the *Drosophila melanogaster* genome. This dataset was processed with the meme application fasta-get-markov, to generate a 5^th^ order hidden markov model of the available background enhancers found in the *Drosophila* blastoderm [40]. This model or its associated fasta file were used as the background model for all relevant processes of the meme suite of applications. A subset of the 401bp sequences, defined by the ftz_3_032707-sym-1 dataset, was used as the positive set for Ftz binding. This set contains 403 intervals identified as bound by Ftz in stage 5 *Drosophila melanogaster* embryos, along with the location of maximal binding within each interval. Genomic sequence for each of these regions was extracted from the repeat masked genome. For both MEME and DREME, a q-value threshold of 0.05 was used with the previously computed background file searching both forward and reverse strands. MEME was restricted to a maximum motif width of 10, while DREME’s default of 8 was used [41]. To search for additional motifs within the enhancer regions, Ftz/Ftz-F1 enhancer regions were analyzed using MelinaII (which runs 4 de novo algorithms, Consensus, MEME, MDScan, and Gibbs) and the *Drosophila* JASPAR database [42]. To search for motifs using the Jaspar database, a Gaussian distribution was constructed from random sequences in the *Drosophila* genome to determine the background likelihood of the occurrence of any given sequence. Enhancer sequences were scanned using pwms from the Jaspar database and compared to this background to determine the chance of a motif occurring randomly at each position. Motifs with a probability score of 0.9 or higher were considered candidates.

## Results

### Microarray identification of Ftz-F1-responsive genes

To identify candidate targets of Ftz-F1, gene expression was compared between wildtype and *ftz-f1* germline clones embryos (referred to throughout as *ftz-f1* mutants) at three time points during development: stage 5, the blastoderm forms, Ftz 7 stripes are established; stage 6, gastrulation, 7 strong Ftz stripes; and stage 8, germband extension, Ftz stripes begin to fade (S1 Fig). Gene expression was compared in whole embryos. As Ftz+ cells represent a maximum of 25% of the cells in the whole embryo, this could potentially dilute the overall fold change observed for genes regulated by Ftz, but this method also allowed for identification of Ftz-F1 targets that are not co-regulated by Ftz.

**Fig 1:**
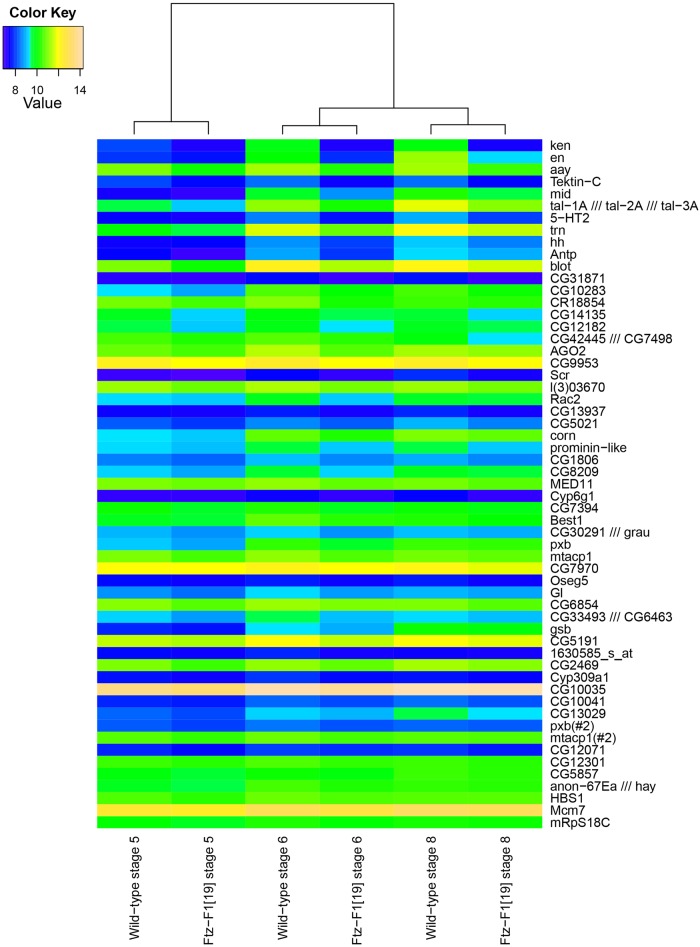
Microarray identification of Ftz-F1-responsive genes in *Drosophila* embryos. RNA was extracted from control or *ftz-f1* mutant embryos at stages 5, 6, and 8, as indicated, and used to synthesize cDNA for hybridization to Affymetrix *Drosophila* 2.0 expression arrays. The dendrogram shows Pearson correlation coefficients of mean expression levels across probesets under assayed conditions. The heatmap shows expression levels of probesets that increased between stages 5 and 6 in wildtype embryos and were expressed at lower levels in *ftz-f1* mutant embryos than control. Genes were sorted from highest to lowest average fold change in expression between control and *ftz-f1* mutants after cellularization.

Of the 18952 probesets on the genechips, 735 (4%) showed detectable alterations in response to absence of *ftz-f1*. To avoid genes with solely maternal expression masking zygotic responses to Ftz-F1, analysis was restricted to genes showing at least a 1.1 fold increase between stage 5 and stage 6 in wildtype microarrays. 3944 (21%) of the probesets showed a 1.1 fold increase in expression level between stage 5 and stage 6. The intersection of these two data sets produced a list of 379 potential targets for validation, fewer than 2% of all genes examined on the microarray. 314 (83%) of these potential targets showed upregulation in response to absence of Ftz-F1 but, since Ftz-F1 has been shown to activate transcription (reviewed in [31], many of these are likely regulated indirectly and were not further examined in this study. Potential direct Ftz-F1 targets were the 65 (17%) probesets that showed at least a 1.1 fold increase in expression level between stages 5 and 6 and a lower expression in *ftz-f1* mutants. These 65 probesets map to 63 unique genes. Fig 1 shows a heatmap of the changes in expression level for potential target genes and S1 Table shows the top 11 candidate target genes, ordered by their fold change in expression level between control and *ftz-f1* mutants. Each showed an average of at least -1.49 fold change in expression level between *ftz-f1* mutants and control embryos. Pairwise comparison of Pearson correlation coefficients showed that individual genechips varied more by developmental stage than by the presence or absence of a functional Ftz-F1 protein (S2 Table). This, along with the fact that fewer than 2% of genes that increase in early zygotic expression show changes in *ftz-f1* mutants, suggests that only a small percentage of genes in the genome are regulated, directly or indirectly, by any given pair-rule transcription factor.

### Expression of Ftz-F1-responsive genes overlaps with Ftz

**Fig 2:**
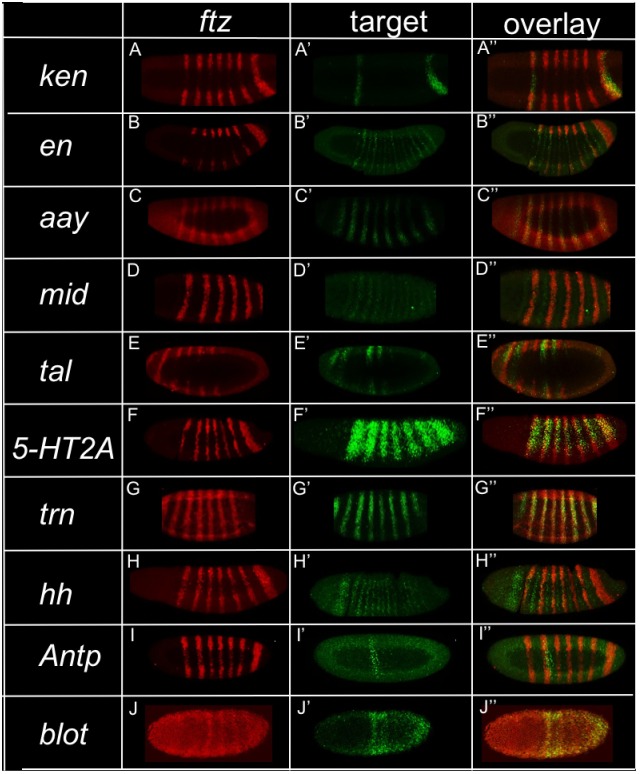
Top Ftz-F1-responsive genes are co-expressed with *ftz*. Fluorescent double in situ hybridization was performed against *ftz* and each target gene, as indicated. *ftz* stripes (A-J, red); target gene stripes (A’–J’, green); overlay (A”–J”, yellow). Some or all of the target genes’ expression patterns overlap *ftz* expression during the blastoderm stage for all candidate targets except *tal*. (A’) *ken*, (B’) *en*, (C’) *aay*, (D’) *mid*, (E’) *tal*, (F’) *5-HT2A*, (G’) *trn*, (H’) *hh*, (I’) *Antp*, (J’) *blot*. Photographs of embryos from confocal microscopy are shown; anterior, left.

All previously identified Ftz-F1 target genes are co-regulated by Ftz. In order to determine whether this is the case for the microarray identified Ftz-F1-responsive genes, expression patterns were examined for ten of the top eleven genes from the microarray and compared to *ftz* expression (Fig 2, Table 1): *ken and Barbie* (*ken*), *engrailed* (*en*), *astray* (*aay*), *midline* (*mid*), *tarsal-less* (*tal*), *5-hydroxytryptamine (serotonin) receptor 2A* (*5-HT2A*), *tartan* (*trn*), *hedgehog* (*hh*), *Antennapedia* (*Antp*), and *bloated tubules* (*blot*). *tektin-C* was not included because it does not show a detectable level of expression in early embryos by in situ hybridization. For 9 of the 10 remaining candidate target genes, expression of the target gene overlapped with *ftz* expression. *ken* is expressed in two stripes, which overlap with *ftz* stripes 1 and 7 (Fig 2A–2A”). *en* is expressed in 14 stripes and every other stripe overlaps with a *ftz* stripe (Fig 2B–2B”). The 7 stripes of *aay* and *ftz* overlap (Fig 2C–2C”). *mid* is expressed in 14 stripes, seven of which overlap with *ftz* stripes (Fig 2D–2D”). Of the six (of a total seven) *tal* stripes visible in blastoderm stage embryos, stripes 2 and 3 are more strongly expressed than the other stripes, but none overlap with *ftz* (Fig 2E–2E”). Thus, *tal* could not be a direct target of Ftz. *5-HT2A* expression is broader than *ftz* expression, but all 7 *5-HT2A* stripes overlap the 7 *ftz* stripes (Fig 2F–2F”). *trn* is expressed in 8 stripes; the 7 posterior stripes of *trn* overlap the 7 stripes of *ftz*, while the most anterior *trn* stripe does not (Fig 2G–2G”). Of the 14 *hh* stripes, 7 alternating stripes overlap with *ftz* (Fig 2H–2H”). The single *Antp* stripe overlaps with the second *ftz* stripe (Fig 2I–2I”). *blot* expression, while in a striped pattern, is not as sharp as other targets. The stripes are blurred and broad, with soft edges. However, this expression does overlap with *ftz* stripes (Fig 2J–2J”). In sum, nine of the eleven top candidate target genes identified in the microarray experiment overlap with *ftz*, *tektin-C and tal* being the exceptions. The former is not detectably expressed and is thus a false positive from the microarray. *tal* may be an indirect target or a gene regulated by Ftz-F1 without Ftz. Overlap for the remaining nine genes was seen in blastoderm or very early gastrulation stages, consistent with the possibility that these genes are regulated directly by Ftz.

**Table 1:** Summary of Ftz/Ftz-F1 dependent target genes and CREs.

Gene	Expression Pattern	Overlap with *ftz*?	Ftz/Ftz-F1 dependent?	Ftz ChIP (1% FDR)	CRE Expression	CRE expression in *ftz*
***en***	14 stripes	Alternate stripes	Yes	Yes	7 stripes (Florence et al. 1997)	Lost (Florence et al.1997)
***ken***	2 stripes	Yes	Yes	Yes	2 stripes	Lost
***aay***	7 stripes	Yes	Yes	No	None	-
***tektin-C***	None	-	-	-	-	-
***mid***	14 stripes	Alternate stripes	Yes	Yes	7 stripes	Lost
***tal***	7 stripes	No	Yes	Yes	-	-
***5-HT2A***	7 stripes	Yes	Yes	No	Weak bands	Lost
***trn***	8 stripes	7 posterior stripes	Yes	Yes	7 stripes	Decreased
***hh***	14 stripes	Alternate stripes	Yes	Yes	14 stripes	Lost
***Antp***	1 stripe	*ftz* stripe 2	Yes	Yes	1 band early, additional late stripes	Decreased
***blot***	Diffuse stripes	Yes	Likely	Yes	Broad early, 7 stripes late	Stripes lost

### Candidate Ftz-F1 target genes require Ftz and Ftz-F1 for expression in embryos

**Fig 3:**
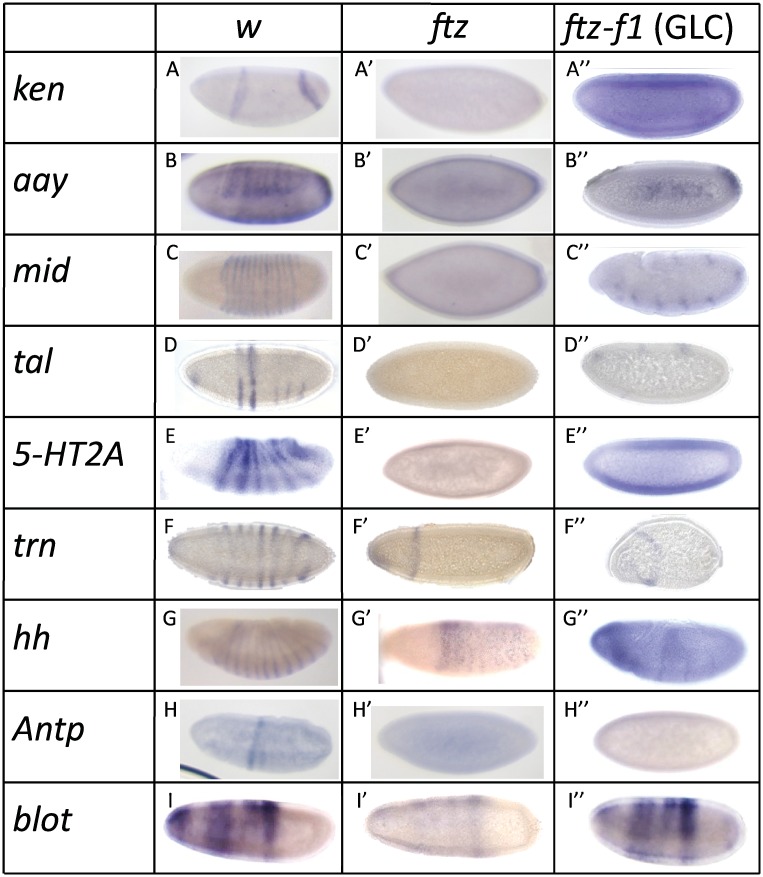
Ftz-F1-responsive genes are regulated by Ftz and Ftz-F1. (A-I) The expression patterns of nine of the top candidate Ftz-F1 target genes from the microarray are shown. All are expressed in stripes in early embryos. (A) *ken*, (B) *aay*, (C) *mid*, (D) *tal*, (E) *5-HT2A*, (F) *trn*, (G) *hh*, (H) *Antp*, (I) *blot*. (A’–I’) Expression was examined in *ftz* mutant embryos. (A”–I”) Expression was examined in *ftz-f1* germline clone (GLC) embryos.

To test whether these candidate target genes are in fact controlled by Ftz and/or Ftz-F1, their expression patterns were examined in *ftz* and in *ftz-f1* mutant embryos (Fig 3, Table 1). *en*, a known target, was not re-examined here [12]. For all nine genes examined here, expression in early stage embryos (Fig 3A–3I) was altered or undetectable in *ftz* (Fig 3A’–3I’) and in *ftz-f1* (Fig 3A”–3I”) mutants. Both *ken* stripes were undetectable in *ftz* and in *ftz-f1* mutants (Fig 3A–3A”). *aay* was expressed in seven stripes in control embryos, which were undetectable in *ftz* and in *ftz-f1* mutants (Fig 3B–3B”). *mid* was normally expressed in 14 stripes. Like *en*, 7 of the 14 *mid* stripes were lost in *ftz* and in *ftz-f1* mutants (Fig 3C–3C”). *tal* expression was undetectable in *ftz* and decreased in *ftz-f1* mutants (Fig 3D–3D”). *5-HT2A* was expressed in seven stripes beginning in early gastrulation in control embryos, which were undetectable in *ftz* and in *ftz-f1* mutants (Fig 3E–3E”). In *ftz* and in *ftz-f1* mutant embryos, the expression of the 7 posterior stripes of *trn* that overlap with Ftz (Fig 2G) was lost while the most anterior stripe, that does not overlap with Ftz, was present (Fig 3F–3F”). *hh* was expressed in 14 stripes, 7 of which were lost in *ftz* and in *ftz-f1* mutants (Fig 3G–3G”). *Antp* was expressed in one band posterior to the cephalic furrow of the embryo, which was lost in either mutant (Fig 3H–3H”). *blot* was expressed in seven diffuse stripes (Fig 3J). Unlike the other candidate targets, the expression pattern was not changed qualitatively but appears weaker in *ftz* and in *ftz-f1* embryos (Fig 3I–3I”).

In sum, the Ftz-F1-responsive genes identified in the microarray that are expressed in early embryos responded similarly to loss of Ftz and to loss of Ftz-F1 (Table 1). This brings to fourteen the Ftz-F1-responsive genes identified to date. All require Ftz for expression in embryos. These genes are *ftz* itself [11], *en* [12], *apt*, *Sulf1* [25], *drm*, *noc*, and *5-HT2A* [26] and seven new targets found in the microarray (*ken*, *aay*, *mid*, *tal*, *trn*, *hh*, A*ntp*). For *blot*, Ftz and Ftz-F1 appear to have a quantitative effect on *blot* expression levels but are not responsible for establishing its striped expression. Finally, the loss of *tal* expression in *ftz-f1* mutants verifies its identification in the microarray experiment. The fact that *tal* does not overlap *ftz* expression (Fig 2E”) suggested that it could be the only Ftz-F1 target identified to date not co-regulated by Ftz. However, since *tal* expression was lost in *ftz* mutants, it is more likely that Ftz and Ftz-F1 work together to regulate *tal* expression indirectly in Ftz^-^ cells. Overall, these findings are consistent with the fact that *ftz* and *ftz-f1* pair-rule phenotypes are indistinguishable, strengthening the conclusion that Ftz-F1 absolutely requires Ftz for its activity in early embryos.

### Identification of candidate Ftz/Ftz-F1-dependent enhancers

**Fig 4:**
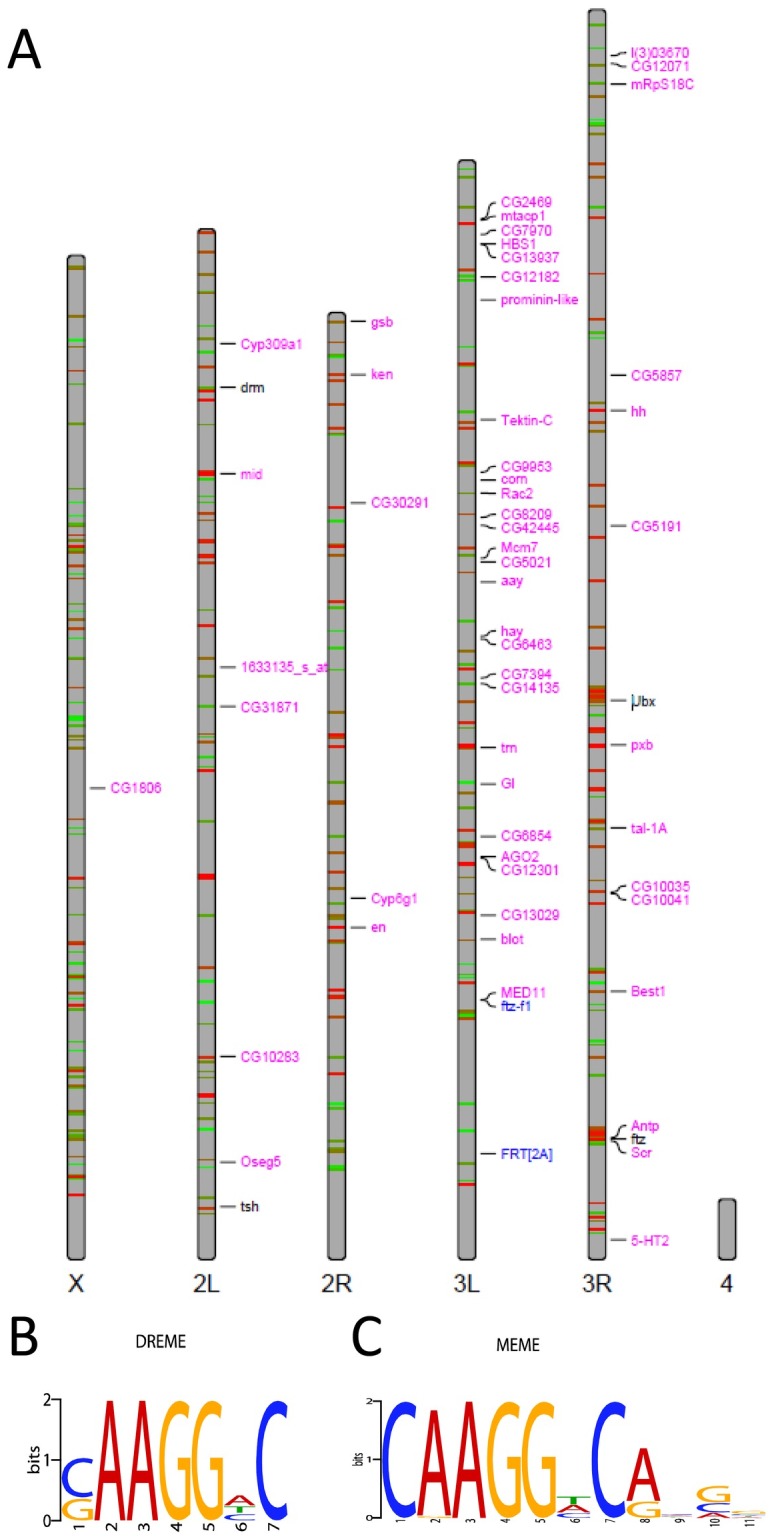
Ftz-F1 binding sites are overrepresented in Ftz genomic binding peaks. (A) Genomic positions of Ftz binding based on Ftz ChIP-chip data from BDTNP is shown schematically mapped to the four *Drosophila* chromosomes [39]. Red lines represent strong Ftz binding; green lines represent weak Ftz binding. The genomic loci of candidate Ftz-F1 targets from the microarray are indicated in pink. *ftz*-responsive genes not found in this study are indicated in black. Positions of *FRT[2A]* and *ftz-f1*, used to generate *ftz-f1* germline clones, are indicated in blue. (B,C) Over-represented sequences in the Ftz ChIP-chip binding data, identified by (B) DREME and (C) MEME. Ftz-F1 candidate binding sites (core, AAGG) are overrepresented in genomic regions where Ftz binds DNA.

The Ftz-F1-responsive target genes analyzed above could be either directly or indirectly regulated by Ftz and/or Ftz-F1. To address this, we made use of BDTNP published ChIP-chip data on Ftz [39] to identify candidate Ftz/Ftz-F1-responsive enhancers within 70 kb each of the top ten Ftz-F1 targets identified from the microarray (S3 Table). Overall, this ChIP-chip experiment identified 403 Ftz binding sites in the genome of blastoderm stage embryos using a 1% FDR (Fig 4A). This cut-off was effective for identifying Bcd target genes [43]. Ftz binding was found using a 1% FDR for eight candidate target genes (Table 1). Genomic regions of ~1 kb surrounding each of these Ftz binding intervals were queried for consensus Ftz-F1 binding sites (BSAAGGHYRHH). At least one candidate Ftz-F1 binding site was found in the region of every Ftz binding peak examined, except for *tal*, for which no Ftz-F1 binding sites were found. DREME and MEME queries of regions within all Ftz binding peaks in the genome identified the core Ftz-F1 binding sequence (AAGG) as the most over-represented sequence (Fig 4B and 4C).

**Fig 5:**
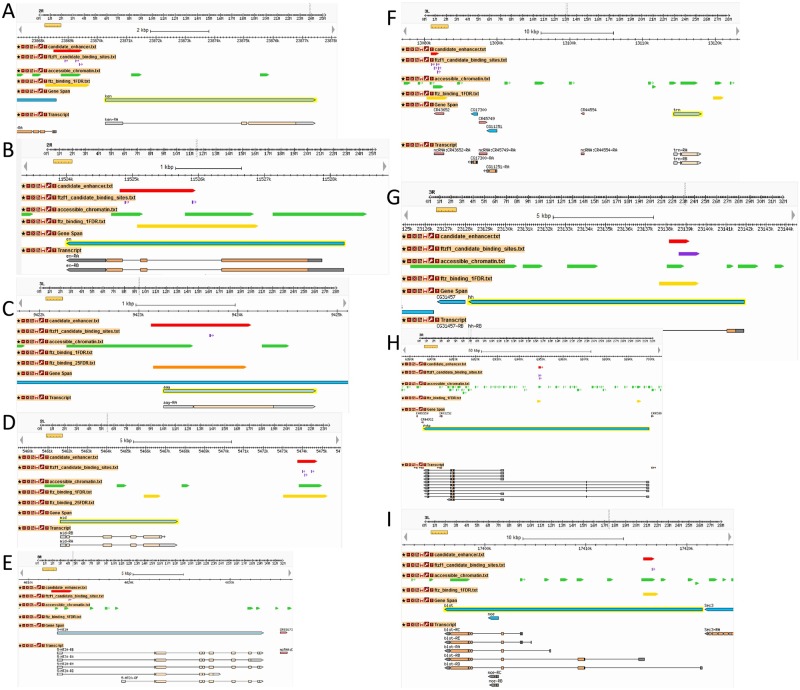
Identification of candidate Ftz/Ftz-F1 dependent enhancers. A screen shot from Flybase GBrowse showing genomic regions around Ftz-F1-responsive genes. Published BDTNP data available at http://bdtnp.lbl.gov/Fly-Net/browseChipper.jsp was downloaded and all interval coordinates along with the peak binding positions were remapped from release 4 to release 6 of the *Drosophila* genome using the Coordinate Converter provided by Flybase. The Ftz binding data was then uploaded into Flybase GBrowse for visualization. Candidate enhancers (red arrows) were identified using three major criteria: 1) Location near Ftz-F1-responsive target gene (light blue); 2) Ftz ChIP-chip binding, yellow arrow (1% FDR) or orange arrow (25% FDR); 3) candidate Ftz-F1 binding sites (thin purple arrows). Open chromatin (green arrows), was also found at blastoderm stage at all enhancers. Enhancer chromosomal locations (flybase v. 6) are: (A) *ken* 2R:23868513..23869466, (B) *en* 2R:11524814..11525958, (C) *aay* 3L:9416225..9417230, (D) *mid* 2L:5473726..5474732, (E) *5-HT2A* 3R:4612279..4614294, (F) *trn* 3L:13073977..13075024, (G) *hh* 3R:23138154..23139164, (H) *Antp* 3R:6948758..6949766, (I) *blot* 3L:17409206..17410251.

For each gene, the region that contained the strongest Ftz binding and at least one candidate Ftz-F1 binding site was selected to be tested functionally (Fig 5). An enhancer was not generated for *en*, even though it was identified in the microarray, because the Ftz/Ftz-F1- dependent enhancer identified previously was also identified by the above searches. Enhancer-reporter constructs generated were: *ken-lacZ*, *aay-lacZ*, *mid-lacZ*, *5-HT2A-lacZ*, *trn-lacZ*, *hh-lacZ*, *Antp-lacZ*, *and blot-lacZ*. For *aay*, a Ftz binding peak was not found in the dataset using 1% FDR but was found at 25% FDR. For *5-HT2A*, no Ftz binding was found. However, because *5-HT2A* was identified as a *ftz/ftz-f1* target here and in a previous study [26], a candidate enhancer was selected based on a candidate Ftz-F1 binding near the gene. In order to increase the strength of this candidate enhancer, an exception for the size of the region was made, making it 2 kb, to include both the strongest potential Ftz-F1 binding sites and Zelda binding sites, which are important in the activation of transcription of many developmental genes [44]. This provided a good test of the importance of Ftz binding in choosing an enhancer. Interestingly, based on BDTNP data, all candidate enhancers were in accessible chromatin regions at stage 5 (Fig 5).

### Candidate enhancers are functional CREs

**Fig 6:**
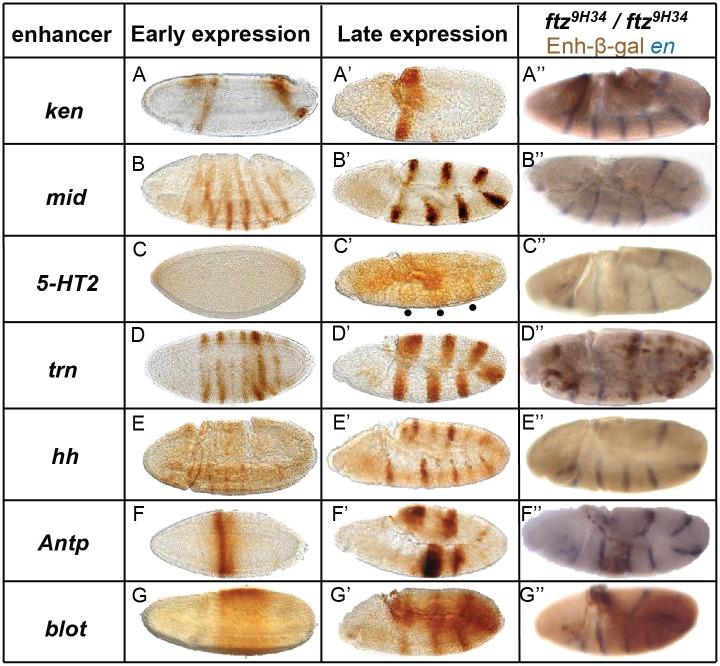
Target gene CREs direct *ftz*-dependent striped expression patterns. Expression of enhancer-*lacZ* reporter constructs, as indicated, in transgenic embryos is shown. (A-G) Early transgene expression. (A’-G’) Late transgene expression. Note that β-galactosidase is stable and accumulates in embryos such that expression appears stronger at late germband extension. Expression of *ken-lacZ*, *mid-lacZ*, *trn-lacZ*, *hh-lacZ*, *Antp-lacZ* and *blot-lacZ* was similar to the corresponding endogenous gene. *5-HT2A-lacZ* was expressed weakly. (A”-G”) Expression of enhancer-*lacZ* reporter transgenes (brown) in *ftz* mutant embryos. To identify *ftz* mutants, in situ hybridization was used to detect *en* (blue), which is expressed in 14 stripes in wildtype embryos and 7 stripes in *ftz* embryos.

To determine whether the candidate enhancers actually function as CREs in vivo, expression of reporter genes was analyzed in transgenic embryos (Fig 6). The *ken-lacZ* reporter gene was expressed in two stripes, anterior and posterior, mimicking endogenous *ken* expression (Fig 6A and 6A’). *aay-lacZ* was the only transgene for which no *β*-galactosidase was detected (not shown). *mid*-*lacZ* was expressed in seven stripes (Fig 6B and 6B’), presumably those that overlap the seven *ftz* stripes (Fig 2D”). *5-HT2-lacZ* was expressed weakly, not evident until germband extension stages when three thick evenly spaced bands were observed (Fig 6C and 6C’). These bands are located between *engrailed* stripes 2 and 3, 4 and 5, and 6 and 7 (data not shown). This weak expression did not correspond to wildtype expression of *5-HT2A*, and the expression was spotty. *trn-lacZ* was expressed in seven of the eight stripes in which endogenous *trn* is expressed (Fig 6D and 6D’). This finding clearly indicates that a Ftz-F1/Ftz-responsive enhancer was identified for *trn*, as only seven of the eight endogenous *trn* stripes overlap with *ftz* [45] and this work). *hh—lacZ* stripes arose during gastrulation, with fourteen stripes evident by germband extension; alternating stripes were strong and weak (Fig 6E and 6E’). The endogenous *hh* gene is expressed in all fourteen stripes at similar levels but only alternating stripes could be regulated directly by Ftz. *Antp-lacZ* was expressed in one strong stripe just posterior of the cephalic furrow at the blastoderm stage, similar to the endogenous *Antp* gene (Fig 6F). Additional stripes appear later, during gastrulation and germband extension (Fig 6F’). *blot-lacZ* was expressed in a broad band in the central region of the embryo at early and late stages (Fig 6G and 6G’) and later in seven stripes, similar to the endogenous expression (Fig 6G’).

In sum, six of the eight enhancers tested directed strong reporter gene expression in patterns virtually identical to the corresponding endogenous genes (*ken*, *mid*, *trn*, *hh*, *Antp*, *blot*), demonstrating that these are functional CREs (Table 1). The two enhancers that were tested despite not fulfilling the criteria used for enhancer identification (see above) were weak or non-functional. *aay-lacZ* was not detectably expressed. This was the one enhancer for which the Ftz ChIP-chip peak was chosen at a 25% FDR. *5-HT2A-lacZ* was expressed but the pattern was extremely weak and differed from the endogenous gene, suggesting that partial regulatory information was present in the region isolated. The *5-HT2A* region chosen was the only enhancer tested that lacked a Ftz ChIP-chip peak but the fact that expression was lost in *ftz* mutants (see below) suggests that there could be low levels of Ftz genomic binding not detected even at 25% FDR. Together, these results suggest that the presence of Ftz binding and the ability to bind Ftz-F1 are highly predictive of enhancer activity in vivo.

### Target gene CREs are Ftz-responsive

To test whether these CREs are *ftz*-responsive, expression was examined in *ftz*^*9H34*^ homozygotes. Embryos were double stained with anti-β-galactosidase antibody (brown) to detect enhancer expression and *en* (blue) to identify the *ftz* mutants, which express only seven *en* stripes. Enhancer-*lacZ* reporter gene expression pattern was examined during germband extension when expression was strongest (Fig 6A”–6G”). The two stripes of *ken-lacZ* expression were lost in the mutants (Fig 6A”). The seven strong stripes for *mid-lacZ* were undetectable in the *ftz* background (Fig 6B”). For *5-HT2A-lacZ*, the one weak enhancer, expression was not detectable in a *ftz* background (Fig 6C”). While some bands were still visible for *trn-lacZ* in *ftz* mutants, these were much weaker and spotty (Fig 6D”), suggesting the presence of additional CRE(s) for *trn* stripes. All fourteen stripes of *hh-lacZ* were lost (Fig 6E”), which was surprising, as only the seven stripes overlapping *ftz* expression should be lost if the enhancer is only under Ftz control; this suggests that this enhancer contains an additional CRE(s) that responds indirectly. Note that this indirect regulation cannot be via *en*, as Ftz regulates only the seven alternate *en* stripes that overlap with Ftz. *Antp-lacZ* expression was much weaker in the *ftz* background (Fig 6F”), suggesting the presence of additional CRE(s). The seven stripes of expression driven by *blot-lacZ* were lost in *ftz* mutants but the broad, central band was still detectable, indicating that this band was not regulated by Ftz. This is consistent with decreased levels of *blot* expression in *ftz* and *ftz-f1* mutants (Fig 3). Overall, seven newly identified enhancers directed expression in embryos in patterns that were weakened or undetectable in a *ftz* mutant background, indicating that these CREs are Ftz-responsive.

### Further analysis of Ftz-dependent enhancers

These Ftz-responsive CREs were next examined for additional motifs that might be important for regulation. For this analysis, the six strong CREs found in this study (enhancers for *ken*, *mid*, *trn*, *hh*, *Antp*, and *blot*) were analyzed along with four previously confirmed Ftz/Ftz-F1-responsive CREs (*ftz* proximal enhancer, *en*, *drm2*, and *drm34* [12, 26, 46] to determine if there were any binding sites common to all ten. These ‘confirmed enhancers’ were compared to ‘false enhancers,’ those that contain Ftz and Ftz-F1 binding sites, but did *not* function as CREs in reporter transgene experiments in vivo. Three false enhancers have been found to date, those for *aay* (this study), *drm1* and *drm5* [26]. These two groups of enhancers were searched independently for binding sites for additional transcription factors that may be necessary regulators of gene expression, either as co-activators/repressors themselves or as factors modulating Ftz/Ftz-F1 DNA binding. All four of the *de novo* algorithms used by the MelinaII program -Consensus, Meme, MDScanner, and Gibbs—identified the binding site for Ftz-F1 as the most common motif in the confirmed enhancers (S2A and S2B Fig). Note that Ftz-F1 sites were also found to be the most common motif in in all Ftz ChIP-chip genomic peaks (Fig 4) suggesting that Ftz and Ftz-F1 binding may be sufficient for target site selection. For the false enhancers, the most common binding motif found was for Forkhead (Fkh) (S2C and S2D Fig).

To determine whether other factors—activating either positively, negatively, or affecting Ftz/Ftz-F1 binding—further influence Ftz/Ftz-F1 target gene regulation, the enhancers were next analyzed to identify known transcription factor (TF) binding sites, using the JASPAR database [42]. Binding sites for 67 known TFs were found in the confirmed enhancers (Table 2). 57 of the 67 TFs contain a homeodomain, as does Ftz, and thus were likely identified by the program because their binding site is similar to the binding site of Ftz. Similarly, this analysis found many homeodomain binding sites in the false enhancers (64 out of 77) as well as 13 TFs that do not contain a homeodomain. Excluding the homeodomain proteins, binding sites for one TF (Zeste) was present in all of the confirmed enhancers but not in all of the false enhancers. Binding sites for 5 TFs (Trithorax-like (Trl, GAF), Scalloped, Dichaete (D), Forkhead, and Sloppy-paired1) were present in all of the false enhancers but not in all of the confirmed enhancers. One TF (DEAF-1) had a particularly high PWM score in both groups of enhancers.

**Table 2:** Transcription factor binding motifs within the confirmed and false enhancer groups.

10 confirmed enhancers		3 false enhancers		10 confirmed enhancers		3 false enhancers	
protein	Homeodomain	protein	Homeodomain	protein	Homeodomain	protein	Homeodomain
br_Z2	n			abd-A	y	abd-A	y
br_Z3	n	br_Z3	n	ap	y	ap	y
br_Z4	n	br_Z4	n	ara	y	ara	y
hb	n	hb	n			bcd	y
Ubx	y			bsh	y	bsh	y
ovo	n	ovo	n	btn	y		
Abd-B	y	Abd-B	y	cad	y	cad	y
Antp	y			caup	y	caup	y
Awh	y	awh	y	ct	y	ct	y
B-H1	y	B-H1	y	ems	y	ems	y
B-H2	y	B-H2	y	en	y	en	y
C15	y	C15	y	eve	y	eve	y
		CG11085	y			exd	y
dbx	y	dbx	y			exex	y
lms	y			ftz	y	ftz	y
		CG13424	y			hbn	y
CG15696	y	CG15696	y	ind	y	ind	y
CG18599	y	CG1859	y	lab	y		
CG32105	y	CG32105	y	lbe	y	lbe	y
CG32532	y	CG32532	y	lbl	y	lbl	y
Vsx1	y	Vsx1	y	mirr	y	mirr	y
CG4328	y	CG4328	y			oc	y
CG7056	y	CG7056	y	otp	y	otp	y
CG9876	y	CG9876	y	prd	y	prd	y
Deaf1	n	Deaf1	n	repo	y	repo	y
Dfd	y			ro	y	ro	y
Dll	y	Dll	y			sd	n
E5	y	E5	y	slbo	n	slbo	n
		Gsc	y	slou	y	slou	y
HGTX	y	HGTX	y	tup	y	tup	y
		Hmx	y			unc-4	y
Lag1	y	Lag1	y	unpg	y	unpg	y
		Lim1	y	vvl	y	vvl	y
Lim3	y	Lim3	y	z	n		
NK7.1	y	NK7.1	y	zen2	y	zen2	y
Oct	n	Oct	n	CG34031	y	CG34031	y
		OdsH	y			D	n
Optix	y	Optix	y			fkh	n
Pph13	y	Pph13	y	H2.0	y	H2.0	y
		Rx	y	PHDP	y	PHDP	y
Scr	y	Scr	y			slp1	n
Six4	y	Six4	y	BEAF-32	n	BEAF-32	n
		Trl	n				

Published ChIP-chip data from 4–12 hours AEL embryos for several chromatin markers, including H3K4me3 and H3K27me3, as well as GAF, PC, and Pho binding [47], was used to analyze regions surrounding the Ftz/Ftz-F1 targets and CREs. For the histone methylation marks, no clear pattern was detected, and in fact, some targets had a strong signal for H3K27me3, a marker for repression, even though they are actively expressed. This could be explained by the broad collection time of the embryos in the ChIP-chip data. While PC and Pho showed no distinct difference between confirmed and false enhancers, strong GAF binding was found only near the three false enhancers. GAF was found near only one of the confirmed enhancers (*ken*), but binding was weak (S3 Fig), suggesting that GAF could repress gene activation by Ftz/Ftz-F1.

## Discussion

This study identified genes regulated by *Drosophila* Ftz-F1 using a microarray that compared expression levels in control and *ftz-f1* mutant embryos (Fig 1). Expression of ten of the eleven top candidate target genes was confirmed in embryos by in situ hybridization; the one false positive from the microarray was *tektin-C*. The remaining ten candidate genes include the previously well-characterized Ftz/Ftz-F1 target, *en* (Table 1). Expression of all ten of these genes responded similarly in *ftz* and *ftz-f1* mutant embryos (Fig 3) and for nine of the ten, expression overlapped with *ftz* (Fig 2). These results demonstrate that, with the one possible exception of *tal*, all identified Ftz-F1-responsive genes also require *ftz* for expression. Candidate enhancers for these nine Ftz/Ftz-F1-responsive genes were identified based on the presence of Ftz-F1 binding sites within a peak of Ftz binding, based upon mined ChIP-chip data (Figs 4 and 5, S2 Fig), including the Ftz-dependent *en* enhancer identified previously. Six of the eight new candidate enhancers directed strong Ftz-dependent reporter gene expression in early embryos that resembled expression of the endogenous gene (Fig 6). Overall, these results confirm mechanistic studies showing that, despite the fact that Ftz-F1 is expressed in all somatic nuclei of blastoderm stage embryos, it absolutely requires Ftz to activate target gene expression in vivo.

### Ftz binding in the *Drosophila* genome

The ChIP-chip data published by BDTNP identified 403 Ftz binding sites in the genome of blastoderm stage embryos using a 1% FDR (Fig 4A). An additional 3,318 sites were identified when a 25% FDR was used as a cutoff. While some of the sites identified at 25% FDR may represent stable Ftz binding, the one tested here (candidate *aay* enhancer) was not functional in directing reporter gene expression. Ftz binding was found in the vicinity of all of the Ftz-F1-responsive genes identified in this study and also near previously identified Ftz-responsive genes not identified in the microarray (Figs 1 and 4A). These include *teashirt* [48], *gooseberry* [48], *Ubx* [49–51], and the *ftz* upstream element [46]. Two Ftz/Ftz-F1-responsive enhancers had been identified for *drumstick (drm)*–*drm2* and *drm34* [26]. Only *drm2* was bound by Ftz in the ChIP-chip analysis—*drm34* was not identified at either 1% or 25% FDR. This is the only known CRE missed in the ChIP-chip analysis, suggesting that a small number of bona fide Ftz target genes were not found with the current data. Within the 403 Ftz binding peaks, the most over-represented sequence was the Ftz-F1 binding site (Fig 4B), suggesting that Ftz-F1 is the primary determinant of stable Ftz binding in the genome. This is consistent with biochemical studies showing that Ftz-F1 dramatically increases the affinity and specificity of Ftz DNA binding in vitro [11, 17].

### Other factors influencing Ftz/Ftz-F1 activity in vivo

Analysis of verified Ftz/Ftz-F1-regulated enhancers (confirmed enhancers), compared to sequences containing potential Ftz and Ftz-F1 binding sites but not functioning as enhancers (false enhancers), was carried out to identify candidate co-regulators that may limit the binding and/or activity of Ftz and/or Ftz-F1 to specific genes. Binding sites for Deaf-1 and Zeste were present in all of the Ftz-dependent enhancers while false enhancers were enriched in binding sites for Dichaete and GAGA Factor. All of these proteins are likely to contribute to the regulation of many early embryonic genes, consistent with their complex phenotypes.

Deaf-1, first identified as a putative cofactor of the Hox protein Deformed, appears to function as a general factor in the early embryo, as mutants display wide-ranging effects on embryonic development including segmentation defects [52]. Zeste binding sites were identified in all confirmed enhancers, and other studies have shown that it can act as both an activator and repressor [53, 54], functioning at the chromatin level [55, 56]. Dichaete and GAF binding sites were both identified in all of the false enhancer sites. Dichaete also affects transcription at the chromatin level Interestingly, it is expressed in stripes in the early embryo [57, 58] and was previously found to interact with Ftz-F1 in a whole genome yeast two-hybrid experiment [59]. Like Zeste, GAF can also act as an activator or repressor on the chromatin level [60], and GAF binding sites have been found in Polycomb response elements (PREs) [61–63], where Polycomb Group proteins (PcG) bind to repress homeotic gene expression, along with Pleiohomeotic (Pho) [64–66]. Examining ChIP-chip binding data of GAF surrounding both the confirmed and false enhancers revealed that GAF bound only at the false enhancers, with the exception of weak binding near *ken-lacZ* (S3 Fig). Together these results suggest that while other TFs may play roles in the regulation of expression of Ftz/Ftz-F1-responsive genes, Ftz-F1 is the main determinant of Ftz genomic binding. In addition, GAF may act as a repressor of Ftz/Ftz-F1 binding.

### Targets of pair-rule genes

This study identified more than 50 genes that are expressed at higher levels in wildtype than *ftz-f1* mutants (microarray). Of the genes showing the largest difference in the microarray experiment, ten of the top eleven are co-regulated by Ftz and Ftz-F1, and identification of enhancers suggests that at least seven of these ten are directly regulated by them. Ftz/Ftz-F1 are PRGs responsible for the formation of even-numbered parasegments and are thought to be among the more downstream PRGs, that, for example, directly regulate *en* expression. However, this study and others suggest that they have many other direct targets.

The new Ftz/Ftz-F1 target genes identified in this study all have known roles in embryonic development. *en* and *hh* play multiple roles, the most relevant to this study being their well-known roles as segment polarity genes [67]. While En is a homeodomain-containing transcription factor, Hh is a signaling molecule. *mid* encodes a T-box transcription factor, classified as a segment polarity gene, but its most well-studied roles are in development in neuroblast specification, axon guidance, and heart development [68–70]. *Antp* is a central *Hox* gene and was one of the first proposed candidate targets of pair-rule genes, including Ftz [71, 72]. This study confirms the proposed direct regulation of Antp by Ftz, along with Ftz-F1. *ken* encodes a zinc finger transcription factor implicated in genitalia development and as a regulator of the JAK/STAT pathway [73–75]. Interestingly, a previous study identified the gap protein Kruppel (Kr) as a negative regulator of *ken* [76], suggesting that a combination of activation by Ftz/Ftz-F1 and repression by Kr could explain the *ken* 2-stripe pattern. *aay* was identified in screens for regulators of peripheral nervous system development [77]. It encodes a putative 3-phosphoserine phosphatase and is necessary for axon guidance in the PNS has a role in axon guidance/nervous system development While initiatilly expressed in a 7-stripe pattern ([78] and Fig 3), it is later expressed in clusters in each segment as well as in the gut [78]. *trn* encodes a cell surface protein that has been implicated in cell movement and migration in various cellular contexts including roles in imaginal discs, tracheal branch outgrowth, retinal epithelial integrity [79–83]. Trn, and its partner Capricious appear to regulate cell-cell interactions in all of these contacts by mediating homophilic cell adhesion. This in turn suggests that Trn may be an important regulator of segment integrity in the early *Drosophila* embryo (unpublished observation). Blot is a putative neurotransmitter with a role in morphogenesis of epithelium [84]. Similarly *5HT2* encodes a serotonin receptor with specific ligand binding with a documented role in convergent extension in Drosophila [85–88].

In sum, of the known Ftz/Ftz-F1targets, excluding *ftz* itself, for which there is strong evidence for direct Ftz/Ftz-F1 regulation, seven encode nucleic acid binding proteins that function as TFs themselves (*en*, *apt*, *drm*, *noc*, *ken*, *mid*, *Antp*) and one is a signaling molecule (*hh*). Five fall into a number of different classes that could implicate them in playing more direct roles in morphogenesis: protein phosphatase (*aay*), serotonin receptor (*5-HT2A*), cell surface protein (*trn*), neurotransmitter transport (*blot*), and sulfatase (*Sulf1*). This suggests that the hierarchy remains regulatory to a large extent at the level of pair-rule genes but also identifies a group of direct targets that may play direct roles in segment formation. However, we suggest that the list of known targets is biased by their strong phenotypes. All of the top microarray targets investigated here were known genes, but the next set (Fig 1) includes many genes for which phenotypes have not been analyzed (CGs). Future experiments will determine whether these genes are also direct PRG targets, contributing to segment formation in subtle ways, such that they had not been previously identified in mutant screens.

## Supporting Information

S1 Fig Ftz-F1 and Ftz protein expression and staging of embryos. (A) Stage 4 (A,E,I), stage 5 (B,F,J), stage 6 (C,G,K), and stage 7 (D,H,L) embryos showing Ftz-F1(green) is maternally expressed and localizes to the nuclei. Ftz (red) reaches its peak level during late cellularization (stage 5), when it is expressed in seven stripes. At the onset of gastrulation (stage 6), the most anterior stripe of Ftz is immediately posterior to the cephalic furrow. The Ftz stripes weaken throughout germband extension (stage 8). Both proteins are co-expressed (yellow) in nuclei of the primordia of even numbered parasegments. (Lower panel) Embryos were collected and hand staged at the times and stages indicated. Samples from each timepoint were immunostained with anti-Ftz antibody to verify stage. Phase contrast microscopy of live, dechorionated embryos in halocarbon oil was used to monitor progress of embryonic development, as shown in bottom panel.
(TIF)

S2 Fig Examination of motifs in candidate Ftz enhancers shows Ftz-F1 as the prominent binding motif. (A,C) Schematic representations of each enhancer across the top of each section, as indicated. Melina II software was used to search for common motifs using 4 algorithms—CONSENSUS, MEME, MDScan and Gibbs, as indicated. The colored boxes represent motifs found by each algorithm common to the group of enhancers queried for (A) the 10 confirmed enhancers and (C) the 3 false enhancers. (B,D) All 4 algorithms identified the most common motif (B) in confirmed enhancers to be the binding site for Ftz-F1 and (D) in false enhancers to be the binding site for Fkh.
(TIF)

S3 Fig ChIP-chip data for Pho, PC, and GAF for confirmed and false enhancers. ChIP-chip data published by Schuettengruber et al., 2009 shows binding by fold change (y-axis) of transcription factors GAF, PC, and Pho along the DNA (x-axis). No discernable difference between Pho and PC binding at confirmed versus false enhancers was evident. GAF bound at all three of the false enhancers but not near nine of the confirmed enhancers, and only weakly near the *ken* enhancer, suggesting it may inhibit activation by Ftz/Ftz-F1.
(TIF)

S1 Table Summary of Ftz/Ftz-F1 dependent target genes and CREs. (DOCX)

S2 Table Transcription factor binding motifs within the confirmed and false enhancer groups. (DOCX)

S3 Table Genomic binding within 70kb of candidate Ftz/Ftz-F1 target genes. (DOCX)
