# Supplementary figures and images for: Activation of Ftz-F1-Responsive Genes through Ftz/Ftz-F1 Dependent Enhancers
Source: PLoS One. 2016 Oct 10;11(10):e0163128. doi: 10.1371/journal.pone.0163128 (PMC5056698; doi:10.1371/journal.pone.0163128)

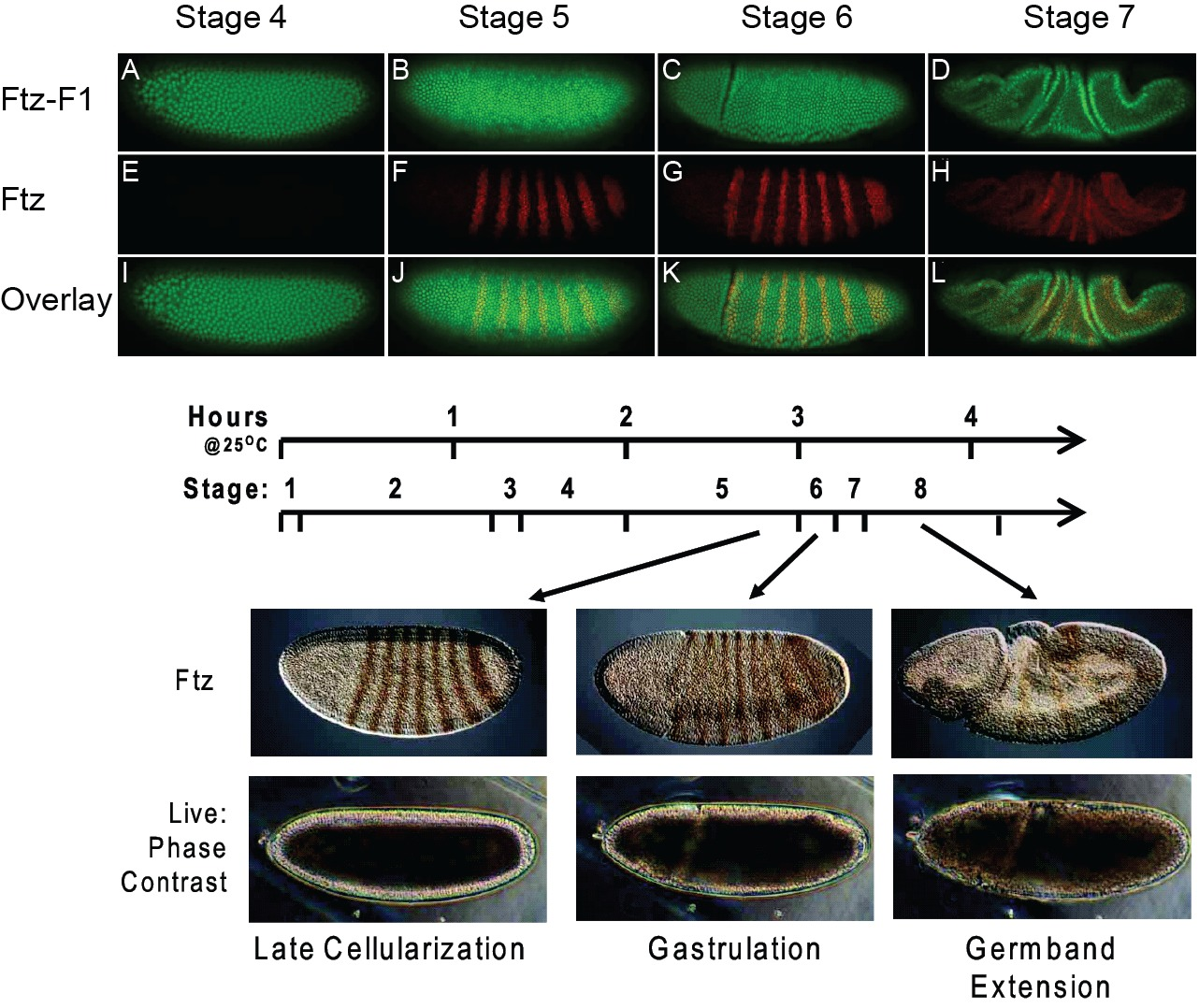

Supplement: S1 Fig — (A) Stage 4 (A,E,I), stage 5 (B,F,J), stage 6 (C,G,K), and stage 7 (D,H,L) embryos showing Ftz-F1(green) is maternally expressed and localizes to the nuclei. Ftz (red) reaches its peak level during late cellularization (stage 5), when it is expressed in seven stripes. At the onset of gastrulation (stage 6), the most anterior stripe of Ftz is immediately posterior to the cephalic furrow. The Ftz stripes weaken throughout germband extension (stage 8). Both proteins are co-expressed (yellow) in nuclei of the primordia of even numbered parasegments. (Lower panel) Embryos were collected and hand staged at the times and stages indicated. Samples from each timepoint were immunostained with anti-Ftz antibody to verify stage. Phase contrast microscopy of live, dechorionated embryos in halocarbon oil was used to monitor progress of embryonic development, as shown in bottom panel. (TIF) [file pone.0163128.s001.tif]

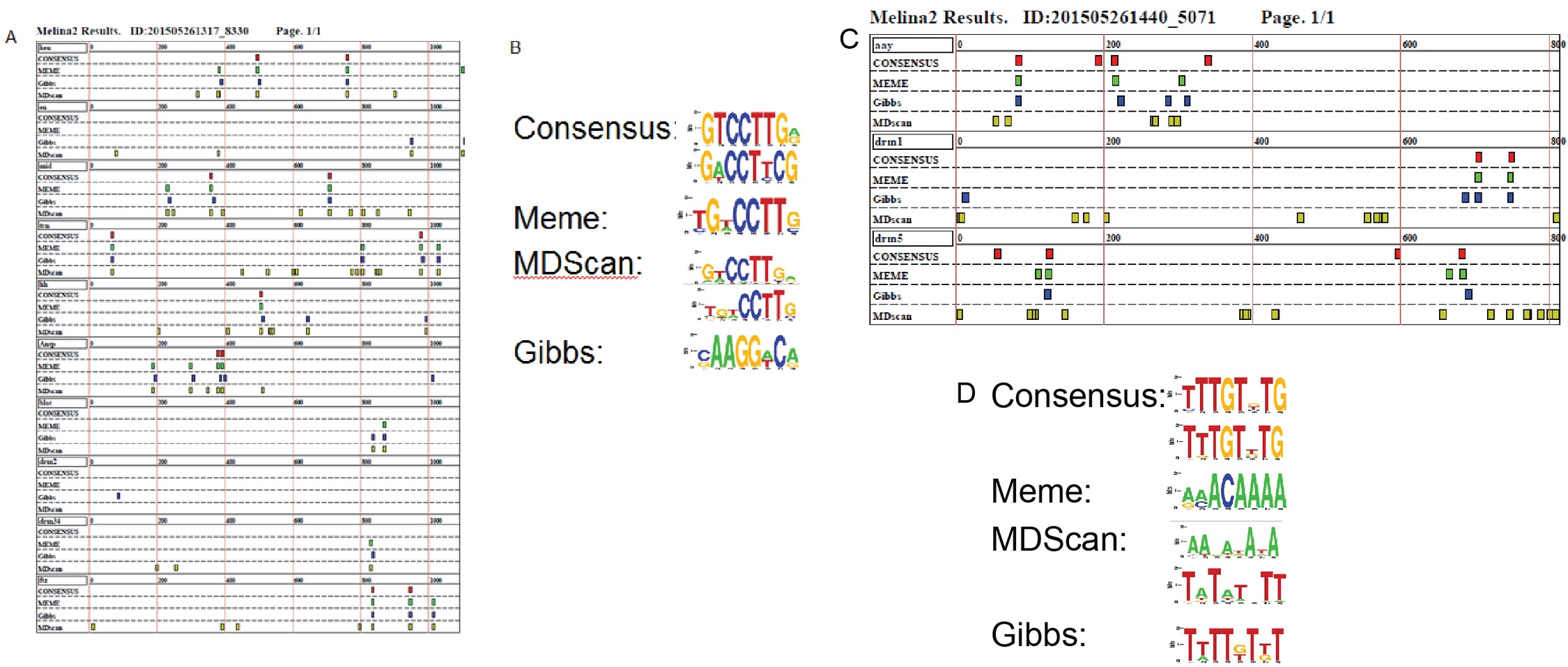

Supplement: S2 Fig — (A,C) Schematic representations of each enhancer across the top of each section, as indicated. Melina II software was used to search for common motifs using 4 algorithms—CONSENSUS, MEME, MDScan and Gibbs, as indicated. The colored boxes represent motifs found by each algorithm common to the group of enhancers queried for (A) the 10 confirmed enhancers and (C) the 3 false enhancers. (B,D) All 4 algorithms identified the most common motif (B) in confirmed enhancers to be the binding site for Ftz-F1 and (D) in false enhancers to be the binding site for Fkh. (TIF) [file pone.0163128.s002.tif]

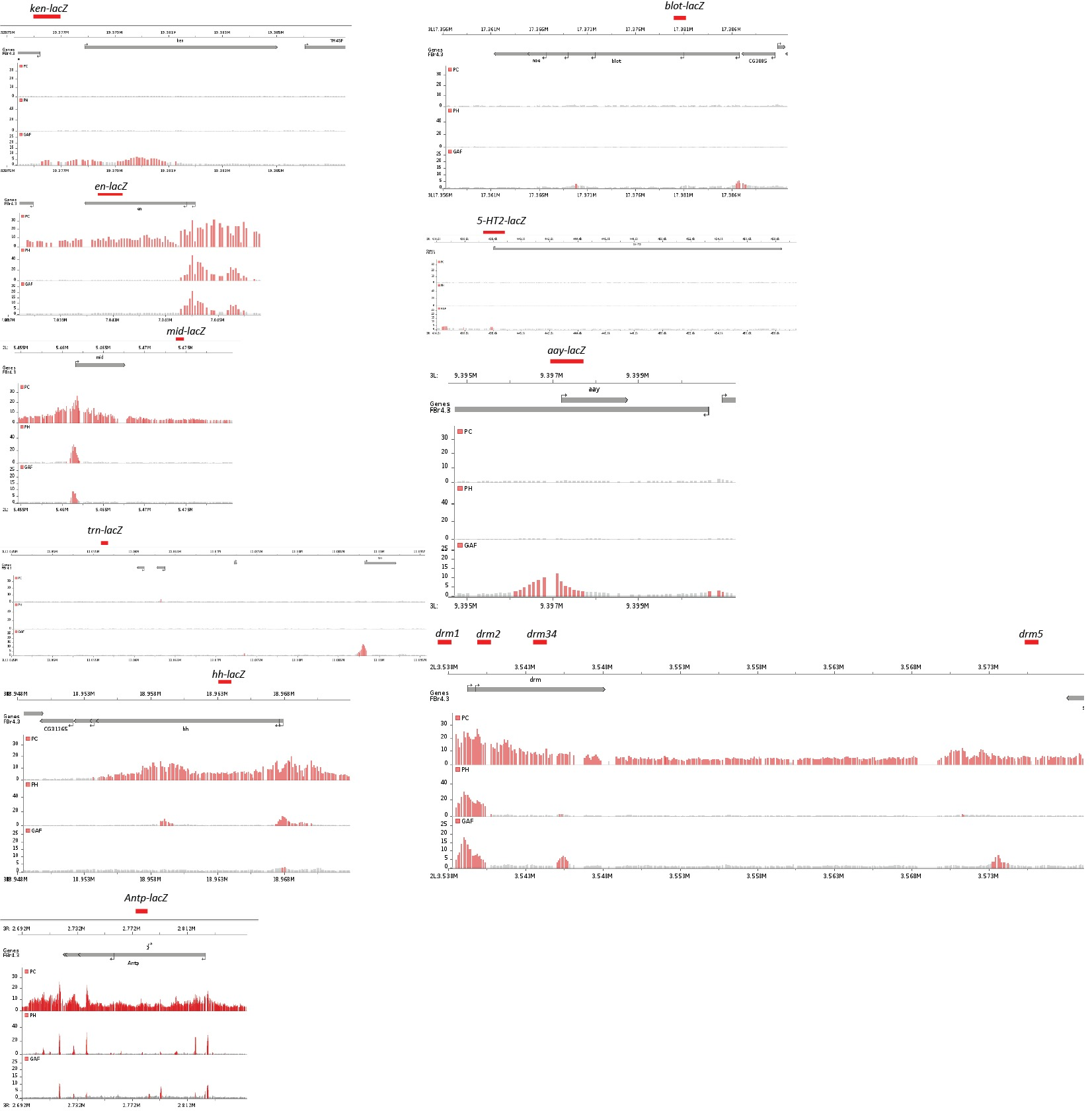

Supplement: S3 Fig — ChIP-chip data published by Schuettengruber et al., 2009 shows binding by fold change (y-axis) of transcription factors GAF, PC, and Pho along the DNA (x-axis). No discernable difference between Pho and PC binding at confirmed versus false enhancers was evident. GAF bound at all three of the false enhancers but not near nine of the confirmed enhancers, and only weakly near the ken enhancer, suggesting it may inhibit activation by Ftz/Ftz-F1. (TIF) [file pone.0163128.s003.tif]

Table S3. Genomic binding within 70kb of candidate Ftz/Ftz-F1 target genes.


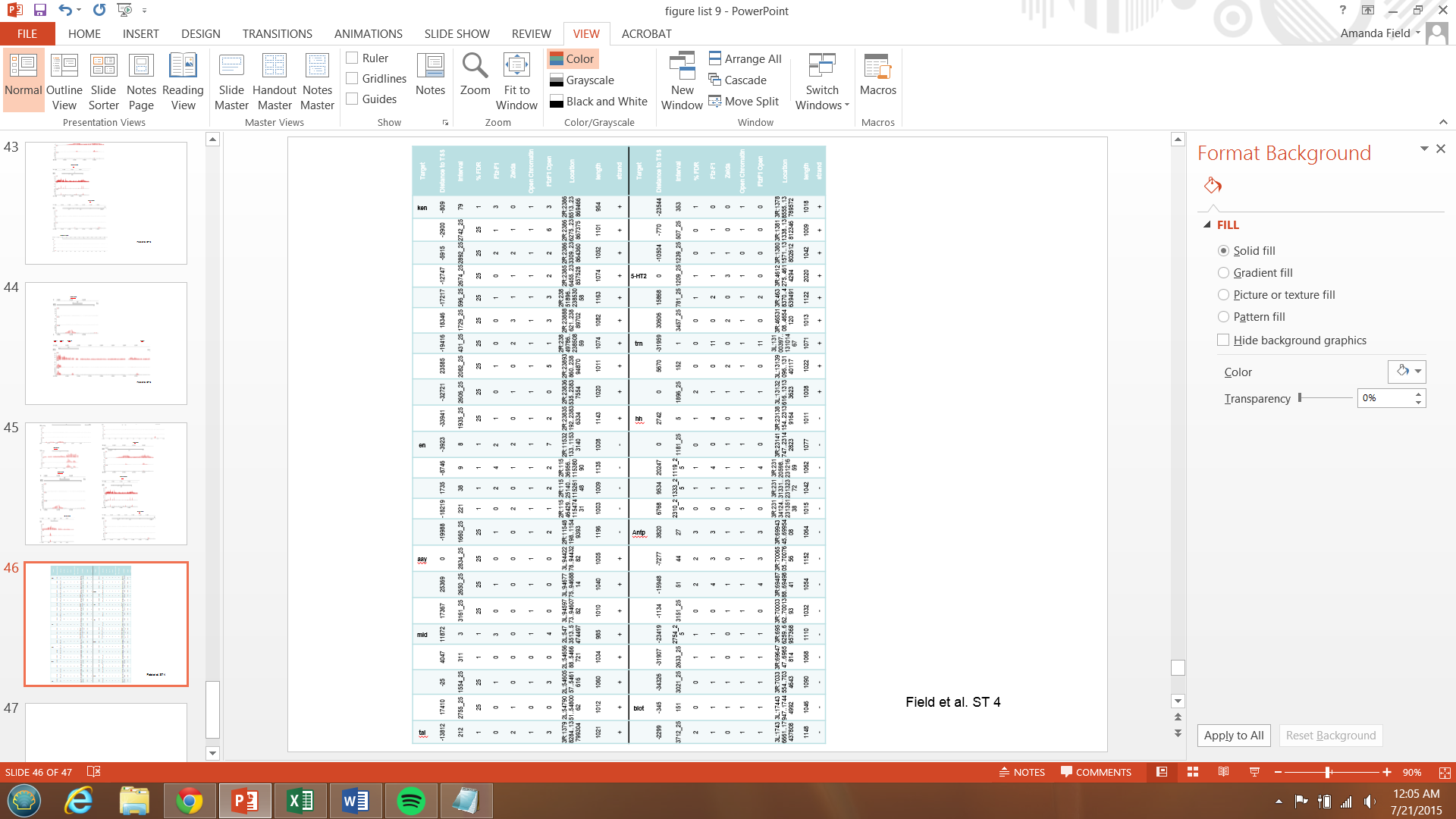

Supplement: S3 Table — (DOCX) [file pone.0163128.s006.docx]
