# Supplementary material for: Activation of Ftz-F1-Responsive Genes through Ftz/Ftz-F1 Dependent Enhancers
Source: PLoS One. 2016 Oct 10;11(10):e0163128. doi: 10.1371/journal.pone.0163128 (PMC5056698; doi:10.1371/journal.pone.0163128)
Supplement: S1 Table — (DOCX) [file pone.0163128.s004.docx]

Table S1. Top eleven candidate Ftz-F1 targets identified by the microarray

| **Gene** | **Affymetrix Probset** | **Mutant Fold Change** | **Stage Fold Change** | **q-value** |
| --- | --- | --- | --- | --- |
| ***ken*** | 1628840_at | -6.1 | 3.43 | 0.00E+00 |
| ***en*** | 1627445_s_at | -4.16 | 4.22 | 0.00E+00 |
| ***aay*** | 1633488_at | -1.89 | 1.22 | 0.00E+00 |
| ***tektin-C*** | 1628238_at | -1.87 | 1.23 | 0.00E+00 |
| ***mid*** | 1637867_at | -1.88 | 5.28 | 2.22E-04 |
| ***tal-1A*** | 1625897_s_at | -1.84 | 3.21 | 0.00E+00 |
| ***5-HT2*** | 1633198_a_at | -1.83 | 2.05 | 6.66E-04 |
| ***trn*** | 1639235_at | -1.69 | 3.32 | 0.00E+00 |
| ***hh*** | 1626527_at | -1.56 | 2.39 | 2.15E-02 |
| ***Antp*** | 1624759_s_at | -1.59 | 2.61 | 6.94E-03 |
| ***blot*** | 1626839_s_at | -1.56 | 2.41 | 0.00E+00 |
