# Supplementary material for: Activation of Ftz-F1-Responsive Genes through Ftz/Ftz-F1 Dependent Enhancers
Source: PLoS One. 2016 Oct 10;11(10):e0163128. doi: 10.1371/journal.pone.0163128 (PMC5056698; doi:10.1371/journal.pone.0163128)
Supplement: S2 Table — (DOCX) [file pone.0163128.s005.docx]

Table S2. Gene expression varies more by developmental stage than genotype

|  | **FF1.5** | **FF1.6** | **FF1.8** | **FRT.5** | **FRT.6** | **FRT.8** |
| --- | --- | --- | --- | --- | --- | --- |
| **FF1.5** | 1 | 0.97114 | 0.947377 | 0.995691 | 0.968568 | 0.946157 |
| **FF1.6** |  | 1 | 0.991669 | 0.96222 | 0.997102 | 0.989425 |
| **FF1.8** |  |  | 1 | 0.937397 | 0.990501 | 0.996754 |
| **FRT.5** |  |  |  | 1 | 0.962672 | 0.939053 |
| **FRT.6** |  |  |  |  | 1 | 0.992129 |
| **FRT.8** |  |  |  |  |  | 1 |
